# Reproducibility of Illumina platform deep sequencing errors allows accurate determination of DNA barcodes in cells

**DOI:** 10.1186/s12859-016-0999-4

**Published:** 2016-04-02

**Authors:** Joost B. Beltman, Jos Urbanus, Arno Velds, Nienke van Rooij, Jan C. Rohr, Shalin H. Naik, Ton N. Schumacher

**Affiliations:** Division of Immunology, The Netherlands Cancer Institute, Plesmanlaan 121, 1066 CX Amsterdam, The Netherlands; Division of Toxicology, Leiden Academic Centre for Drug Research, Leiden University, 2333 CC Leiden, The Netherlands; Genomics Core Facility, The Netherlands Cancer Institute, Plesmanlaan 121, 1066 CX Amsterdam, The Netherlands; Center for Chronic Immunodeficiency (CCI), University Medical Center Freiburg and University of Freiburg, Freiburg, Germany; Walter and Eliza Hall Institute of Medical Research, 1G Royal Parade, Parkville, VIC 3052 Australia; Department of Medical Biology, The University of Melbourne, Parkville, VIC 3010 Australia

**Keywords:** Cellular barcoding, Lineage tracing, Next generation sequencing, Sequencing error, PCR error, Illumina

## Abstract

**Background:**

Next generation sequencing (NGS) of amplified DNA is a powerful tool to describe genetic heterogeneity within cell populations that can both be used to investigate the clonal structure of cell populations and to perform genetic lineage tracing. For applications in which both abundant and rare sequences are biologically relevant, the relatively high error rate of NGS techniques complicates data analysis, as it is difficult to distinguish rare true sequences from spurious sequences that are generated by PCR or sequencing errors. This issue, for instance, applies to cellular barcoding strategies that aim to follow the amount and type of offspring of single cells, by supplying these with unique heritable DNA tags.

**Results:**

Here, we use genetic barcoding data from the Illumina HiSeq platform to show that straightforward read threshold-based filtering of data is typically insufficient to filter out spurious barcodes. Importantly, we demonstrate that specific sequencing errors occur at an approximately constant rate across different samples that are sequenced in parallel. We exploit this observation by developing a novel approach to filter out spurious sequences.

**Conclusions:**

Application of our new method demonstrates its value in the identification of true sequences amongst spurious sequences in biological data sets.

**Electronic supplementary material:**

The online version of this article (doi:10.1186/s12859-016-0999-4) contains supplementary material, which is available to authorized users.

## Background

Single cells are the fundamental units that determine the formation and behavior of multicellular organisms, and there is a growing interest to understand the kinship and differentiation potential (‘fate’) of single cells. For example, how do individual cells reach the locations where they exert their function? How much do individual cells differ in the amount and type of offspring they produce, and how does this give rise to the behavior of the entire population of cells?.

Technologies that have been developed over the past years now make it feasible to follow in vivo cell fate. As a first example, intravital imaging can be used to track stationary cells over time scales of days to weeks, to reveal how much offspring individual cells produce over time and whether such offspring sticks close together in space. In this manner, direct evidence for the existence of cancer stem cells has recently been obtained for several cancer types by means of intravital imaging [1–4]. As an alternative approach to monitor single-cell behavior, and that is also compatible with migratory cell types, unique genetic markers of individual cells may be used to follow their progeny. Early work in this area employed the analysis of viral integration sites [5, 6] but integration site recovery bias precludes highly quantitative analyses by this method. To circumvent this issue, an alternative strategy called cellular barcoding was developed in more recent years [7–16]. In this approach, individual cells are tagged with unique DNA barcodes at a specific time point. These barcodes are passed on to all offspring of cells and at a later point in time the type and amount of offspring of each barcoded cell can be determined. Spatial information at a within-tissue level is typically not available in cellular barcoding data, but a comparison between tissues and between different cell types within a tissue is feasible. Quantification of the amount of offspring of a barcoded cell is achieved by PCR amplification, followed by next generation sequencing. Indexing of samples allows one to run many samples of different cell types, organs and time points within a single deep sequencing run, thereby allowing high-throughput acquisition of data [17].

A non-trivial problem in the quantification of cellular barcoding data is the relatively high rate of PCR and sequencing errors. Such errors lead to spurious sequences which, when not filtered out, can alter the conclusions with respect to lineage relationships, and in particular with respect to the number of founder cells that contribute to a cell population of interest. Without any filtering, the majority of NGS reads derived from a single highly abundant barcode is correct. For example, for a uniform error rate of 0.001 (0.1 %) per base pair, the expected fraction of correct reads for a nucleotide stretch of 100 equals (1–0.001)^100^ ≈ 0.90. However, because for each erroneous sequence the position and the substitution of the error(s) are different, the vast majority (>99 %) of individual sequences that is detected is spurious [16]. For instance, considering only variants with one nucleotide difference from a single stretch of 100 nucleotides, there are already 300 possible erroneous variants.

To help distinguish true barcodes from error-derived barcodes, the library of barcodes that is used for cell labeling can be independently sequenced to generate a reference library of barcodes that can potentially be encountered during subsequent experiments. Nevertheless, because also upon amplification and sequencing of a barcode library errors will occur, such a reference library is prone to contain a proportion of erroneous sequences. Furthermore, in situations in which the diversity of the barcode library is very high, the generation of an accurate reference library is precluded by attainable sequencing depth. As a more fundamental limitation, in experimental settings in which barcodes are generated de novo, for instance through induced DNA recombination or inversions [10, 18], the development of a reference library is inherently precluded. Also in other NGS applications, such as determination of T cell receptor or immunoglobulin repertoires, of heterogeneous viral populations, and of cancer cell populations, spurious sequences need to be filtered out without the help of a reference library [15, 19–22].

The most commonly used approach to deal with sequencing errors is to merge closely related sequence variants [23–25], as it seems likely that these variants emerged from a ‘mother’ sequence. However, this can lead to the inadvertent removal of true sequences that happen to show sequence similarity to another sequence present. To improve the detection of sequencing errors, novel PCR approaches have recently been developed in which individual DNA molecules receive unique tags [26–29], or that rely on ‘circle sequencing’ [30]. It has been shown that such approaches can be used to detect very rare sequence variants and this may also apply to cellular barcoding data. However, the complicated nature of these techniques may reduce barcode detection efficiency when individual barcodes are present at low numbers. As an alternative way to improve sequence error detection, coding theory may be used to design barcode libraries with sufficient distance between the individual barcodes [31–33], after which the barcodes can be synthesized one by one. For example, using barcodes of 10 nucleotides long, more than 7000 barcodes that have a Levenshtein distance of at least three (i.e., three substitution or indel events are required to have one barcode be misread as another barcode) can be generated [31].

Here, we examine how the nature of sequencing errors in NGS data can be used to improve the distinction between true and erroneous sequences. Analysis of experimental barcoding data sets from an Illumina HiSeq platform shows that the many sequencing errors that are found in such NGS data occur at a frequency that is predictable across the different samples within a sequencing lane. We exploit this finding by developing a novel approach to remove spurious sequences from cellular barcoding data that is based on a combination of sequence similarity and predictability of the frequency of a particular error. We subsequently apply this method to several barcoding data sets and show that it results in very accurate estimates of the true barcodes in biological samples.

## Methods

### Cellular barcoding data

We re-analyzed raw NGS data from several previously described cellular barcoding experiments [10, 11]. First, a training data set was utilized that contained 20 Jurkat cell clones labeled with distinct barcodes that had been previously identified by Sanger sequencing [10]. These cell clones were mixed into a single base sample in cell numbers diverging in two-fold steps from the smallest to the largest clone (i.e., by mixing ≈ 10 cells from clone 1, ≈20 cells from clone 2, and so on, up to ≈ 5.2x10^6^ cells from clone 20). A second base sample was created with the same series of clones in reverse order of prevalence. Both clone mixes contained ≈ 10.5x10^6^ cells in total, and these base samples were then diluted in a series of ten-fold steps (i.e., 50, 500 and 5000), and in a series of two-fold steps (i.e., 500, 1000, and so on until ≈ 1x10^6^), resulting in expected cell numbers ranging from ≈ 10 to ≈ 2.1x10^5^ cells per sample for all input clones combined. Subsequently, samples were equally divided into two parts to create technical replicates, and these replicates underwent PCR amplification and were analyzed by NGS. Note that Gerlach et al. [10] showed that the input cell numbers for the known barcodes correlated well with the corresponding read abundances, although small clones had a tendency to be underrepresented in terms of read numbers.

Based on this training data set we developed a cleaning procedure (see below) that we subsequently applied to two experimental data sets. In one of these data sets, barcode-labeled naïve CD8^+^ T cells were injected into recipient mice receiving *Listeria monocytogenes* infection, after which offspring was analyzed at various time points after infection or reinfection (10). In the other experimental data set, barcode-labeled lymphoid-primed multipotent progenitors (LMPPs) were injected into partially irradiated recipient mice, after which progeny (e.g. monocytes, dendritic cells, B cells, neutrophils) was analyzed following several weeks of proliferation and differentiation [11]. In all experiments, each sample was split into two technical replicates of equal size and each independently underwent a PCR to amplify DNA and to attach a sample index (note that these sample indices were designed such that they have a Hamming distance of at least two nucleotides when compared to any of the other indices). Dozens to hundreds of samples were pooled and sequenced on an Illumina HiSeq 2000 platform. Detailed descriptions of the experiments are given in [10, 11].

### Procedure to detect spurious sequences

Raw next generation sequencing data were processed as follows: First, from the reads that contain an exact match to a (constant) part of the sequenced primer region, the sample index and 15 nucleotides of the barcode were extracted based on the relative position with respect to the detected primer region. Barcodes were then divided over the corresponding sample indices in that sequencing lane, requiring an exact match to one of these indices. A table of read counts was constructed that contained, for each (unfiltered) barcode, the number of reads for each of the samples. This table served as input to the below described algorithm that removes spurious sequences.

In order to decide whether a barcode could be derived from a particular mother barcode, three properties of sequence pairs were determined: (i) Their Levenshtein distance *d*_*L*_ [34], (ii) the ratio of the ‘total frequencies’ of the two barcodes (least prevalent divided by most prevalent, i.e., *r* = ∑*c*_*i*_*/*∑*m*_*i*_, where *r* is the ratio, *c*_*i*_ is the number of reads of the least prevalent barcode in sample *i* and *m*_*i*_ is the number of reads of the most prevalent barcode in that sample), (iii) the predictability of the relative frequencies of a given sequence pair in individual samples within a sequencing lane. To quantify the latter property, the ratio of the total frequencies of a pair was used to predict the expected frequencies for the individual samples, i.e., the expected number of reads for sample *i* equals *r · m*_*i*_. Due to the stochastic nature of sequencing errors, the variation around this expectation should be greatest for small values. To take this into account, we used a beta-binomial distribution to assign a probability to the observed read numbers of each sample. Given *c*_*i*_ observed reads in sample *i* of the least prevalent barcode and *m*_*i*_ observed reads in the corresponding sample of the most prevalent barcode, the probability of this observation then equals *f(c*_*i*_*,m*_*i*_*,*α*,*β*)*, where *f* is the probability density function for the beta-binomial distribution with shape parameters α and β. This can be re-parameterized to a ‘mean’ μ and ‘overdispersion’ parameter ρ by setting μ = α/(α + β) and ρ = 1/(1 + α + β). Using the latter parameterization, we fixed μ to the ratio of the total frequencies *r* and ρ to *r*/10, because this gave rise to a unimodal distribution with a peak at the expected number of reads for the least prevalent sequence in sample *i*. The ‘log-likelihood score *l*’ was subsequently calculated by taking the mean of the natural logarithms of the probability densities for each sample in which either *m*_*i*_ or *c*_*i*_ has at least 200 reads. The data points that did not fulfill this requirement are excluded because we observed that, even for clearly correct mother-daughter pairs, at these low read numbers it occasionally happened that a daughter sequence had more reads than a mother sequence in only one of the samples, which would negatively affect quantification by the log-likelihood score. A threshold log-likelihood score was defined depending on the total number of reads of the daughter barcode, according to *l* = *a* log10(∑*c*_*i*_) + *b*, where *l* is the log-likelihood score and *a* and *b* are the slope and offset of the threshold line. Pairs with a score above the threshold qualified as a mother-daughter pair if the other requirements were also fulfilled. The parameters used for these requirements were: *d*_*L*_ ≤ 4, *r* ≤ 0.05, *a* = −2, *b* = −1.

With the above objective definition of mother-daughter pairs considered correct, we first sorted all unfiltered barcodes based on their total number of reads over all samples. Barcodes with a combined total of less than 100 reads in all samples of the lane were excluded because a proper discrimination between correct and incorrect mother-daughter pairs is precluded by the low numbers of reads per sample (for the fifty to hundreds of samples in our data only a handful of reads per sample is expected). In addition, barcodes with such a low abundance are likely to include daughters with more than four nucleotide differences from their mother (the applied threshold), and these daughters would escape detection without the additional read threshold. Barcodes with ‘N’ values at one or more nucleotide positions were also excluded. For the remaining barcodes, the most prevalent sequence was compared to all other sequences and for the pairs that fulfilled the three criteria of correct mother-daughter pairs, the daughter barcodes were removed. Subsequently, the next most prevalent barcode was compared to all remaining less prevalent barcodes, again removing all barcodes considered as daughters. This procedure was continued until all barcodes were either removed because of likely mother-daughter relations or kept as true barcodes. Note that the clean-up approach does not alter the abundance of retained barcodes because reads from spurious barcodes are removed rather than joined to their mother barcode. The algorithm was implemented in R (see Additional files 1 and 2). The running times of the algorithm were on the order of minutes on a single Xeon-E3 1225 processor, 3.20 GHz, with 16 GB of memory (e.g., for our biggest data set about four minutes).

## Results

### Overview of experimental barcoding technology

**Fig. 1:**
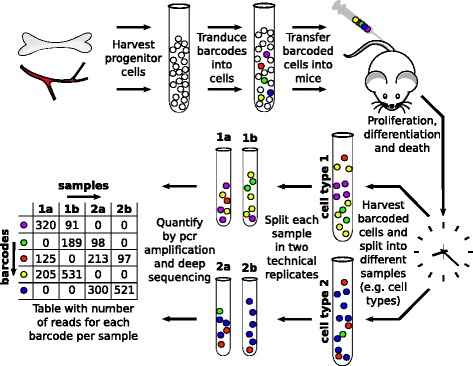
Overview of experimental barcoding technology and barcode quantification. In brief, progenitor cells isolated from organs (e.g. bone marrow) are labeled with unique, heritable barcodes (represented by differently coloured cells). Barcoded cells are injected into animals, after which cellular proliferation, differentiation and death occurs. Different cell types are then harvested, DNA is extracted, and the resulting samples are split into technical replicates. These undergo PCR amplification and deep sequencing, resulting in a table with the number of reads for each barcode in each sample

In cellular barcoding (Fig. 1), progenitor cells of interest are isolated from appropriate tissue and exposed to a library of retro- or lenti-viral vectors that each carry one DNA barcode from a large pool (usually thousands) of barcodes. Infection leads to the incorporation of the DNA barcodes into the host cell genome. The barcoded cells, recognizable because they for instance express GFP from the introduced genome, are then transferred back into a host. Alternatively, barcodes may be induced in vivo by recombination of a synthetic recombination substrate (10). During the subsequent time period, the labeled cells pass on their barcode to all offspring. After a period of days – months, during which the cells can proliferate and differentiate, progeny cells are isolated from the host to quantify each barcode within offspring cell populations of interest.

In our procedures, each sample (i.e., a cell population of interest) is split into two equal-sized technical replicates in order to be able to determine the reproducibility of the subsequent barcode quantification (note that this is not standard procedure in every lab). For each technical replicate, DNA is extracted and amplified by PCR. During one of the amplification steps, each technical replicate also receives a sample index such that many samples can be multiplexed during NGS. Finally, next generation sequencing using the Illumina HiSeq 2000 platform is performed on the amplified DNA products. The number of reads that are retrieved for each barcode in a specific sample is roughly proportional to the number of cells that carried that barcode within the cell sample [10]. Thus, the amount of offspring for each barcoded cell can be estimated. Comparison of barcode reads between technical replicates can be used to assess the accuracy with which the barcode repertoire within a given cell population is described. Comparison of barcode reads in cell populations that are by definition not derived from the same progenitor (e.g. isolated from different animals) can be used to determine random background overlap in barcode repertoires [17, 35].

### Read thresholds are insufficient to remove spurious barcodes

During PCR amplification and next generation sequencing, errors are frequently generated, either leading to erroneously amplified barcodes that are sequenced or to correctly amplified barcodes that are erroneously sequenced. Errors that occur early in PCR amplification are rare and it is therefore unlikely that the same early PCR error occurs in both technical replicates. Thus, such PCR errors can be filtered out by removing barcodes that differ greatly in frequency between the two corresponding technical replicates. Such a strategy also removes barcodes that have ended up in samples by infrequent 'physical' contamination, because also in this case one and the same contaminating sequence is unlikely to occur in both replicates. However, this strategy will not remove most sequencing errors because these are more abundant than PCR errors, implying that the same error is likely to occur in both replicates. Likewise, when DNA template molecules accumulate during PCR amplification, ‘late PCR errors’ may occur in corresponding technical replicates. In this work, we focus on strategies to remove errors that occur at similar frequencies in technical replicates, and that can therefore not be filtered out by removing barcodes that differ greatly in frequency between the two corresponding replicates.

Although the error rate of sequencing is higher than that of early PCR mistakes, it is on average still less than one percent on a per-nucleotide basis, and a natural thought may therefore be that spurious sequences will generally only have few reads. In such a case, it could be sufficient to apply a read threshold, possibly based on an estimate of the number of cells in a sample, to filter out erroneous sequences. Indeed, this approach may work well when all barcodes occur at approximately equal levels. However, it was recently shown that even for such a ‘uniform-occurrence’ scenario, a frequency threshold is not a perfect solution [16]. More importantly, for the typically large variation in the amount of offspring of individual progenitors in biological samples [10, 11], this problem is expected to be worse. In order to investigate whether a straightforward read threshold is of value when used on heterogeneous data, we re-analyzed a barcoding data set with a large, artificially generated, variation in barcode abundances, and in which the approximate clone sizes for each barcode was known [10]. Specifically, this data set was composed of cell clones each containing one out of 20 known barcodes, and highly diverse numbers of these cell clones were mixed into a base sample, which was diluted by different factors. The resulting samples were subsequently divided into two equal parts and the technical replicates underwent PCR amplification and were analyzed by NGS on an Illumina HiSeq platform.

**Fig. 2:**
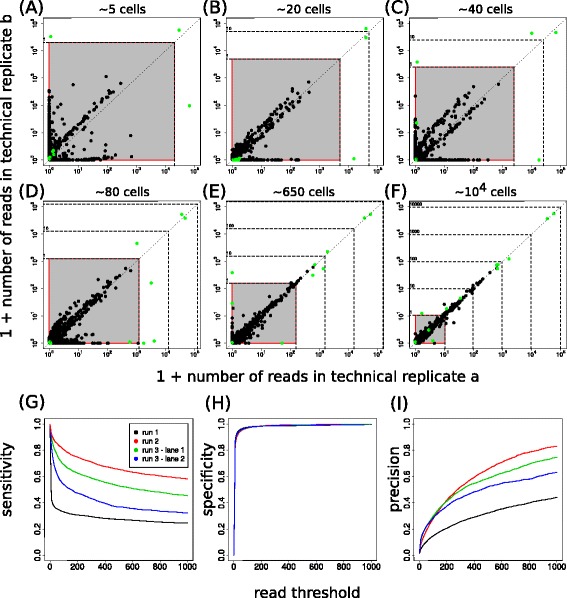
A read threshold is insufficient to remove spurious barcodes. **a**-**f** Experiments with 19 clones of known barcodes, mixed in different frequencies and then diluted such that the expected total cell number per technical replicate varies from ~5 cells to ~10^4^ cells for all clones combined. Plots show number of reads in each of two technical replicates after normalization to 10^5^ reads per replicate. Green dots denote barcodes that were true, black dots denote spurious barcodes. The grey region with red border approximates a cell count of one or less, i.e., the frequency of barcode reads within this range is below what is expected for a single cell. Dashed horizontal and vertical lines and numbers alongside denote the approximate number of cells to which the normalized read numbers correspond. **g**-**i** Quantification of the performance of filtering based on a fixed read threshold (without prior normalization) when considering reference-list-based filtering as a gold standard, applied to barcode sequencing data on T cell differentiation (8) and haematopoiesis (9). Sensitivity (**g**), specificity (**h**) and precision (**i**) are shown as a function of the applied read threshold for four sequencing lanes (denoted by the different colors)

Because in this set-up the identity of true barcodes is known (in this case 19 barcodes, as one of the input barcodes was not found back), true and erroneous sequences in the NGS data can be distinguished. In order to determine the read count of spurious barcodes relative to true barcodes, plots were generated in which the total read count for each barcode was visualized for corresponding technical replicates (Fig. 2a–f, each dot represents one barcode). As expected, true barcodes (depicted as green circles) usually either stay above the approximate read threshold expected for a single cell (i.e., stay outside of the grey regions), or are clearly part of the noise because they only have a handful of reads, potentially due to sample index errors. Note that true barcodes can end up on one of the two axes when derived from low cell numbers, because the splitting into two technical replicates can lead to zero cells in one of the replicates. For samples with low cell numbers (up to ≈ 50 cells per technical replicate), heterogeneity in detected barcode clone sizes is limited, and erroneous variants have read numbers below what is expected for a single cell. This indicates that in a setting with little variability in clone size, an appropriate read threshold, based on total cell numbers within a sample, can remove noise without substantially affecting the detection of true barcodes. Importantly though, in samples with more divergent clone sizes, read counts of spurious barcodes and true barcodes partially overlap, making it impossible to distinguish true and false barcodes based on just read frequency.

In order to investigate whether clone size heterogeneity also reduces the value of a simple read threshold for the level of heterogeneity found in biological samples, we analyzed two previously generated data sets that describe single cell output in antigen specific T cell responses [10] and single cell output of LMPPs [11]. In the original analyses, only those barcodes that were considered part of the independently sequenced reference library were retained for further analysis. Although this reference library may miss some true barcodes and may contain a small amount of spurious sequences, it provides a good approximation of the barcodes actually present in an experiment. Therefore, we compared the sequences that were considered true or spurious after either read-threshold-based filtering or after filtering against the barcode reference library. Using the reference-library-based filtering as a gold standard, we calculated the sensitivity (probability of ‘correctly’ identifying a barcode as true), the specificity (probability of ‘correctly’ identifying a barcode as spurious), and the precision (fraction ‘correctly’ identified barcodes amongst all barcodes left after filtering). Plots of these performance measures as a function of the applied read threshold (Fig. 2g–i) show that a large fraction of spurious barcodes can be filtered out because most have very few reads (high specificity). However, the number of spurious barcodes left remains substantial (low precision) and many true barcodes are incorrectly filtered out (low sensitivity). In conclusion, in the case of large clone size heterogeneity as is typical for biological samples, filtering of barcode sequencing data using a straightforward read threshold is inadequate.

### Consistency of sequencing error frequency across samples

In the technical replicate plots for the experiments that used known barcodes, we noticed that in many cases the noise in the data formed approximately straight lines (Fig. 2a–f). In some cases, this phenomenon manifested itself as finger-like structures, with a true barcode being detected at the same ratio but at higher read counts (Fig. 2b–d). Moreover, only in cases in which a true barcode occurred on one of the axes (implying absence of that barcode in one of the technical replicates, likely due to unequal division over the replicates), noise was also detected along that axis (e.g. compare Fig. 2b and f). These observations strongly suggested that a single line of noise in a technical replicate plot (diagonal or along an axis) can be caused by errors derived from a single barcode. Indeed, the sequences at such a single line of noise were in nearly all cases similar to the corresponding, higher abundant true sequence (Additional file 3). The observation that these errors form a single line implies that the errors occur at almost the same frequency in associated technical replicates. In other words, for individual errors, the error rate is reproducible between replicates.

**Fig. 3:**
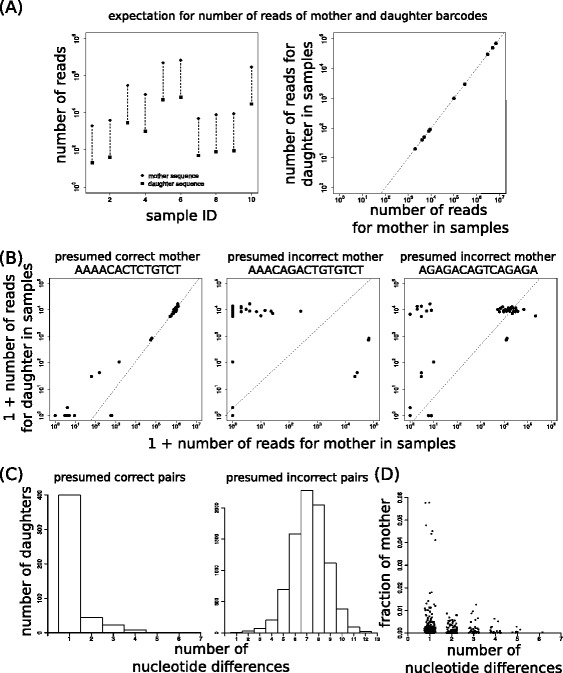
The frequency of sequencing errors is highly predictable across samples within a single lane. (**a**) Artificial data from a hypothetical sequencing lane with read numbers for one mother barcode and an associated daughter barcode, derived by sequencing error, in ten samples. Read numbers for mother and daughter barcode are either plotted as a function of sample ID (*left panel*) or against each other (*right panel*). **b** Examples of the read counts of three potential mother barcodes and of one particular spurious barcode plotted against each other, for presumed correct (*left panel*) and incorrect (*other panels*) mother-daughter pairs. Each dot represents one technical replicate, lines denote the prediction based on total frequencies of mother and daughter barcodes. Note that only for one of the pairs the frequency of errors is quite predictable across the samples, strongly suggesting that the spurious barcode derives from that mother. **c**, **d** The 500 most frequent spurious barcodes were compared to all 19 mother barcodes and the presumed mother was selected based on predictability of sequencing errors across samples by visual inspection. The number of nucleotide sequence differences was determined for each presumed mother-daughter pair (**c**, *left panel*) and for every other possible pair (**c**, *right panel*). For the presumed mother-daughter pairs the fraction of reads of the daughter sequence relative to the mother sequence was also determined (**d**)

We investigated whether such reproducibility of the rate at which one particular error occurs extends towards other samples in the same sequencing lane. To visualize this, consider a hypothetical sequencing lane in which one of the true barcodes occurs in ten samples and has a different frequency in each of them. If sequencing errors occur at a reproducible rate, in each sample one particular daughter barcode would be generated with a constant percentage of reads of the mother barcode (left panel in Fig. 3a – dashed lines connect mother and daughter, in this hypothetical example always differing by a 100-fold). Such a constant mother – daughter relationship can also be visualized by plotting the read numbers of potential mother and daughter barcodes in different samples against each other, which is expected to approach a straight line (right panel in Fig. 3a).

To investigate whether this visualization helps to detect spurious barcodes in our data set with 19 known barcodes, in Fig. 3b we consider one particular spurious barcode which is compared to the possible true barcodes (in this example 3 out of 19 shown). In only one case a straight line is clearly approached (left panel), thereby identifying the presumed mother barcode. We extended this analysis by making similar read count plots for the read numbers of the 500 most frequent spurious barcodes, comparing to the read counts of the 19 true sequences across the different samples. For the vast majority of spurious sequences (474 out of 500 cases), a single potential mother could be identified for which both the number of reads was related in a linear manner with that of the spurious sequence in each sample, and for which the sequences were similar. A comparison of the number of nucleotide differences between presumed correct and incorrect mother-daughter pairs revealed that on average the identified mother-daughter pairs were much more similar than incorrect pairs (Fig. 3c; mean ± SD 1.6 ± 1.0 versus 8.2 ± 1.5 nucleotide differences). Moreover, the read counts of spurious sequences were only a small fraction of their mother sequence read count and exceptional cases with a somewhat higher frequency (up to 6 %) occurred only infrequently (Fig. 3d). Thus, the rate at which a particular sequencing error occurs is not only very similar for technical replicates, but this observation extends to all samples in which a given barcode occurs. In conclusion, although sequencing errors occur randomly and their frequency varies substantially between different errors, the frequency with which a particular error occurs is quite predictable within a sequencing lane.

**Fig. 4:**
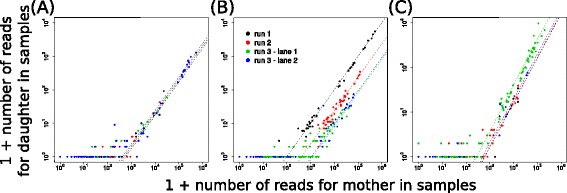
Predictability of the frequency of sequencing errors in complex biological samples. **a**-**c** Examples of the number of reads of presumed mother and daughter sequences plotted against each other. Each dot represents one technical replicate, lines denote the prediction based on total frequencies of mother and daughter barcodes. Colors denote in which run or lane a sample was sequenced. Examples are shown for pairs which have an approximately equal error frequency across runs and lanes (**a**), which have different error frequencies across runs yet similar frequencies across lanes of the same run (**b**), and which have different error frequencies across lanes of the same run yet similar frequencies across runs (**c**)

The observed predictability of sequencing errors could potentially be a peculiarity of the artificially generated data set that contained only 19 true barcodes. To address this, we subsequently investigated whether this phenomenon also occurred in biological samples with a much higher complexity. To this purpose, we used the unfiltered data from two previously published barcoding data sets [10, 11] on T cell output (two sequencing lanes in different runs) and LMPP output (two sequencing lanes in the same run). These experiments used the same library of viral barcodes, so some barcodes are expected to occur in samples in all four lanes. We selected several of such barcodes that occurred with high abundance in all lanes and within the unfiltered data, we searched for barcodes that differed by only one base from these sequences, and were therefore likely to represent sequencing errors. Subsequently, we compared the frequencies of these presumed spurious barcodes to those of their mother sequences in all technical replicates (Fig. 4, colours represent different lanes). As might be expected, exactly the same sequencing errors could occur in different lanes or runs. The frequency at which particular errors occurred could differ by up to ten-fold between lanes (Fig. 4b–c). Importantly though, also in this setting, the relative frequency of each spurious barcode was very reproducible across biological samples within a single lane. Hence, although the frequency of particular errors can be variable across sequencing lanes or runs, within a single lane it is highly predictable.

### A novel approach to filter out spurious sequences

The observation of conserved mother-daughter ratios within sequencing lanes opens up the possibility to exploit this feature for the detection of sequencing errors. For that purpose, an objective score is required to decide whether a barcode should be considered to be an error from a potential mother barcode or not. We designed such a score based on the total number of reads of a barcode (i.e., by summing the reads for that barcode for all samples in the lane) relative to the total number of reads of a potential mother. The fraction of these two totals can be used to predict the expected read number of the daughter sequence for each individual sample, based on the read number for the mother sequence in that sample. For instance, consider a mother with in total 1,000,000 reads and an associated daughter, derived by sequencing error, with in total 1000 reads (i.e., a fraction of 0.001). For a sample with 100,000 reads for this mother sequence, the expected number o0066 reads for the daughter sequence is then 0.001 × 100,000 = 100. Because errors are generated by a stochastic process, deviations from this expectation occur and should be larger for samples with few reads than for samples with many reads.

**Fig. 5:**
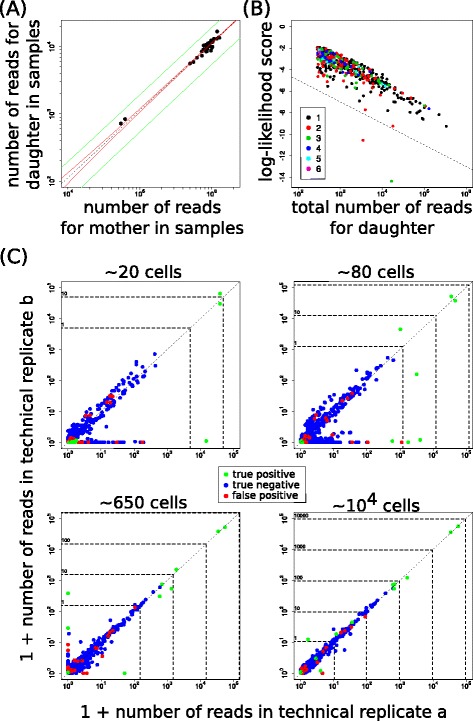
Predictability of sequence error frequency allows for detection of spurious barcodes. **a** Example of zoom-in of read numbers of potential mother and daughter sequences plotted against each other. Each dot represents one (half)-sample, dashed black line denotes the prediction based on total frequencies of mother and daughter barcodes, solid lines denote 95 % confidence band when assuming that errors are described by a binomial distribution (*red*) or a beta-binomial distribution (*green*). **b** ‘Log-likelihood score’ of presumed mother-daughter pairs as identified by visual inspection, as a function of the total read number of the daughter barcode. Each dot denotes one pair and its color denotes their number of nucleotide differences. Dashed line represents the threshold above which pairs are subsequently considered correct. **c** Result of cleaning procedure on data with different dilutions of 19 known barcode clones (expected cell numbers per technical replicate denoted above panels). Dots represent read number in each of the two replicates, colors denote whether the barcode was a true positive, a true negative, or a false positive. Note that there are no false negatives in this simple data set. Dashed horizontal and vertical lines and numbers alongside denote the approximate number of cells to which the normalized read numbers correspond

We first used the data set with 19 known barcodes to show that the variation in the read numbers of spurious sequences around the expected values could not be described by a binomial distribution, as many observations fall outside of the 95 % confidence band (red lines in Fig. 5a, this figure is the same as the left panel Fig. 3b with zoom-in). To model this clear overdispersion in the data, we switched to a beta-binomial distribution (confidence band in green lines in Fig. 5a) that is frequently used to capture large variation [36, 37]. For each comparison of a barcode with a potential mother barcode, we calculated a ‘log-likelihood score’, based on the probabilities of observing the read numbers of each sample according to a beta-binomial distribution with fixed shape parameters (for details see Methods). We applied this score to combinations of mothers and spurious sequences that were presumed correct or incorrect based on visual inspection of plots as in Fig. 3b (i.e., leading to the pairs used in Fig. 3c–d). The score decreased in a linear fashion when plotted as a function of the logarithm of the total number of reads of the daughter barcode (Fig. 5b). However, sequence similarity has no general impact on the log-likelihood score as is apparent from the mixing of the colors in Fig. 5b (which denote the number of nucleotide differences between mother and daughter barcodes). Importantly, a comparison of the scores obtained for mother-daughter pairs revealed that incorrect pairs typically had scores different from correct pairs, especially for similar sequences (Additional file 4). We used this information to define a cutoff line (dashed lines in Additional file 4) such that only a small number of outlier mother-daughter pairs would be missed, i.e., the cutoff line approximately separates correctly and incorrectly assigned mother-daughter combinations. Because this separation was best for similar sequences (Additional file 4), we used sequence similarity as an additional criterion to assign mother-daughter combinations (at most four nucleotide differences). Moreover, because the total frequency of a barcode relative to its mother sequence is typically low (Fig. 3d), we included relative frequency as the third criterion (at most 5 %).

Thus, our 'training' data set provided detailed information on how to set thresholds for log-likelihood score, sequence similarity and relative frequency in order to recognize spurious barcodes that are generated from a mother sequence by sequencing errors. On the basis of these requirements, we constructed a ‘clean-up’ algorithm that starts with the most frequent barcode in a lane and compares it to all other barcodes in that lane, in order to identify and remove likely daughter barcodes. Subsequently, the next most frequent remaining barcode is considered as a potential mother and compared to all barcodes occurring at a lower frequency. This cycle is continued until all barcodes had been considered as potential mothers. When applied to the data set with known barcodes, the clean-up procedure removed none of the 19 true barcodes (Fig. 5c, green dots) yet was able to filter out 94 % of the incorrect barcodes (blue dots) that together made up 93 % of the erroneous reads. Part of the remaining spurious barcodes were only observed in one out of two matching technical replicates, suggesting that they are in part sample contaminations or PCR errors that occurred in only one of the replicates, and that can be filtered out by an additional reproducibility filter step. The spurious barcodes that remained after filtering and that were frequent in at least two corresponding technical replicates were more than the employed threshold of four nucleotide differences apart (Fig. 3d). We decided not to increase this threshold further because this would lead to a reduced sensitivity in case of more complex biological samples (see below). In conclusion, the observation that sequencing error frequency is predictable across samples allows one to identify and remove the majority of spurious barcodes in a ‘training’ data set with known barcodes.

### Filtering of spurious sequences in complex cellular barcoding data

**Fig. 6:**
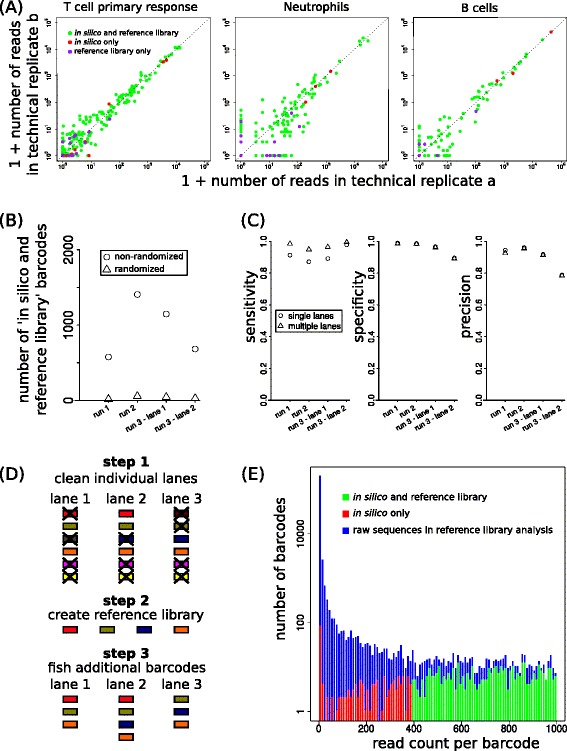
Ability to distinguish true and spurious barcodes in complex data sets. **a** Examples of results of clean-up procedure on barcoding data from four different sequencing lanes on T cell differentiation and haematopoiesis. Dots represent read numbers in each of the two technical replicates and their colors denote whether the barcode was identified as true by both the *in silico* clean-up procedure and by reference-library-based filtering, was in-silico-identified only or reference-library-identified only (barcodes filtered out by both approaches not plotted). **b** Number of barcodes left after cleaning that are also present in the reference library, either with or without prior randomization of both the barcodes and the samples. **c** Comparison of clean-up procedure to the barcodes that are true according to the reference list of the viral barcode library. Considering the reference list as a gold standard, the sensitivity (*left panel*), specificity (*middle panel*) and precision (*right panel*) are shown for the clean-up algorithm for each of the four individual lanes (denoted by ‘single lanes’), and when using the *in silico* created reference library based on the use of the clean-up algorithm on the separate lanes (denoted by ‘multiple lanes’). **d** Sketch explaining the concept of constructing an *in silico* reference library that can be used to combine information from multiple lanes during cleaning. Each colored symbol denotes a distinct barcode. **e** Histogram of the number of reads per barcode in the independent sequencing of the barcode reference library, after zoom-in on infrequent barcodes. Barcodes occurring in the experimental data (at least one of the four lanes) are highlighted in green and red

The training data set contained only 19 true barcodes, a complexity that is much lower than in typical biological applications. Thus, it was important to evaluate whether this novel barcode cleanup procedure also performs well on complex data sets with a variety of barcodes obtained from different cell populations. To this purpose, we applied the clean-up procedure to the sequencing lanes on T cell and LMPP output from [10] and [11] using the same cutoff parameters as above, and compared the result to that of reference-library-based filtering. To visualize whether there was agreement between the two filtering approaches, we made plots of technical replicates (see examples in Fig. 6a) in which we coloured barcodes with agreement in green (‘*in-silico-*and-reference-library’), barcodes only identified as true by our clean-up procedure in red (‘*in-silico-*only’) and barcodes only identified as correct by the reference library approach in purple (‘reference-library-only’). This visualization demonstrates that there is very large agreement between the barcodes considered true by the two approaches. To estimate how much of this agreement could have arisen by chance despite being based on a very different selection procedure, we applied the same cleaning procedure to data in which both the barcodes and the samples were randomized. Specifically, we first randomly swapped the barcode sequences, then assigned each sample read number to a random sample for each individual barcode, and applied the cleaning procedure on these randomized data. Following such randomization, reference-library based filtering is obviously not altered (as each sequence remains unchanged). However, the frequency and sequence similarity relationship between mothers and daughters – the basis for the approach developed here – is lost. Notably, analysis of the resulting data showed that the number of true positives following randomization was only about 3–4 % of that obtained without randomization (Fig. 6b), demonstrating that the large agreement between the two approaches is not primarily due to chance.

To quantify the added value of the clean-up algorithm more precisely, we calculated sensitivity, specificity and precision for each of the four sequencing lanes, while considering the reference library as a gold standard (Fig. 6c). Both sensitivity, specificity and precision were typically higher than 0.9, something that could not be achieved with just a read threshold (Fig. 2g–i), and implying that our clean-up approach is very good at separating true and spurious sequences. We also varied the maximal sequence similarity allowed for mother-daughters pairs presumed correct (Additional file 5) and the offset of the log-likelihood threshold line (Additional file 6). This sensitivity analysis showed that a threshold of three or four sequence differences gave a good separation of true and spurious barcodes for all four sequencing lanes. The performance of the log-likelihood cutoff was very sensitive on one side only. Still, the value chosen based on the training data set (dashed line in Additional file 6) was approximately optimal for all sequencing lanes, demonstrating the general applicability of the log-likelihood approach.

A limitation of our clean-up approach may be the requirement that barcodes need to occur in multiple samples within the same sequencing lane. The data from our complex biological samples consist of fifty to hundreds of samples per lane and therefore fulfill this requirement. In order to study how many samples are needed for a good performance of our approach, we used the complex barcode data to generate artificial data with fewer samples per data set. Specifically, for each lane we randomly selected fixed numbers of samples and normalized the remaining reads such that the total was the same as in the original lane. Cleaning up these artificial data demonstrated that precision was affected by low sample numbers but that a precision of 0.8–0.9 could typically be obtained with as few as 10–20 samples (Additional file 7). This number should be seen as a rule of thumb and will depend on how frequently barcodes overlap between the different samples. Given that multiplexing of samples before sequencing is already common practice because of cost efficiency and will become even more so as read numbers delivered by sequencing equipment increase, we expect our clean-up approach to be of use for many (future) data sets.

Barcodes that are true yet incorrectly identified as spurious in one sequencing lane might also occur in other lanes and be correctly identified as a true barcode in that case. It may be possible to ‘rescue’ such barcodes by combining information of multiple lanes (similar to an approach used by Lu et al. [9]). We therefore constructed a clean-up-algorithm-based barcode reference library: First, the cleanup procedure was applied to all individual lanes (Fig. 6d, step 1). Second, we constructed an *in silico* reference library by joining all barcodes that remained after cleaning in at least one of the individual lanes (Fig. 6d, step 2). Third, we used this *in silico* constructed reference library to fish for additional true barcodes in the separate lanes (Fig. 6d, step 3). We tested whether this approach further improved the performance of the clean-up procedure while not introducing much more *in-silico*-only barcodes. Indeed, sensitivity increased substantially for most lanes, whereas specificity and precision were hardly affected (Fig. 6c).

When comparing the sequenced reference library with the in silico constructed reference library, overall sensitivity was ≈ 0.94, specificity was ≈ 0.97 and precision was ≈ 0.85. In the original sequencing of the library a conservative threshold was used to select ‘true’ barcodes. Thus, it could be that some of the barcodes identified in our clean-up but not in the reference library are in fact true barcodes. To test whether this was possible, we analyzed whether the *in-silico*-only barcodes were also identified during original sequencing and if so, how many reads were obtained. To visualize this, we created histograms in which the number of barcodes having a certain abundance can be seen (Fig. 6d, for example, barcodes with up to 10 reads occurred almost 100,000 times). Histograms were overlaid for (i) all barcodes in the raw data (blue bars), (ii) the barcodes that were part of the reference library and also detected by our *in silico* approach (green bars) and (iii) the barcodes that were only detected by our *in silico* approach (red bars). Almost 200 of the approximately 340 ‘*in silico*-only’ barcodes were also present in the original sequencing of the library and ≈ 100 of these had more than 50 reads (Fig. 6e, red bars). This suggests that some of these sequences will indeed be true barcodes that were filtered out upon the independent sequencing of the barcode library. Together, these results show that our clean-up approach based on predictability of sequencing error frequency can detect true barcodes in complex biological samples.

## Discussion

Next generation sequencing of PCR-amplified DNA is an extremely valuable tool in biomedicine, to diagnose disease, to discover its causes and to assist in the development of new treatments [38–40]. The bioinformatic analysis of such high-throughput sequencing data needs to solve non-trivial problems, for example during the assembly of whole genomes from many short read fragments [41]. One of these problems is to filter out sequencing errors. This problem is particularly difficult when polymorphic regions are sequenced, especially when relevant sequences can occur at low frequencies. For example, T cell receptor (TCR) and Immunoglobulin repertoires are highly diverse and contain many low-frequency sequences, which makes the distinction between true and spurious sequences a difficult problem. In the analysis of such data, sequences with only few nucleotide differences are typically joined, assuming that they are likely to originate from the same mother sequence [15, 19, 20]. However, a consensus on how to best deal with this problem has not been reached [23]. Similarly, to determine somatic mutations in cancer genomes, healthy and tumor tissues within individual patients are compared [21]. Copies of mutated DNA molecules may again be rare, e.g. because only a small fraction of tumor cells may carry a specific mutation. As a third example, viral quasispecies can also be highly heterogeneous and can consist of many low-frequency viral variants, as for example in the case of HIV [42].

Also in cellular barcoding, sequences that are generated by PCR or sequencing errors can severely complicate lineage tracing. A straightforward approach to filter barcoding data for such errors is to apply a frequency threshold [9, 12, 43], which can potentially be based on total cell numbers or on cell numbers of spiked-in clones. However, in many biological systems, the clonal burst of individual cells can vary over orders of magnitude [10, 11] and spurious barcodes derived from highly prevalent barcodes may therefore occur at a similar frequency as true barcodes from lower frequency cell clones. Indeed, consistent with a recent report [16], we show that it is insufficient to filter cellular barcoding data based on just a read threshold (Fig. 2).

A second approach to filter cellular barcoding data is to make use of a reference library that is obtained by sequence analysis of the entire barcode library at an early stage, e.g. when a library of viral vectors has been created [10, 11]. When vectors carrying different barcodes each have an approximately equal abundance, it is expected that sequencing of their DNA to a sufficient depth leads to a clear distinction between true and spurious barcodes. In practice, this distinction is however not that apparent, due to biases in the input DNA sequences or to variation introduced during viral vector growth, leading to an over-representation of some barcodes (e.g. Fig. 6c). Thus, even after application of a conservative threshold to define a reference library it is likely that some true barcodes are missed and some spurious barcodes are included. Moreover, for very large libraries of tens/hundreds of thousands of barcodes, sequencing of the entire library gives insufficient depth to cover all true barcodes. Finally, in systems in which barcodes are generated de novo by individual cells themselves [10, 18] rather then introduced, creation of a reference library is by definition precluded.

Here, we present a novel approach to clean up barcoding data that does not require independent sequencing of a reference library, and that is based on our observation that individual sequencing error occurs at a predictable rate across samples in Illumina HiSeq data. Based on this finding, we devised a strategy to filter out spurious sequences, which represents a useful alternative to employing an independently sequenced reference library for filtering experimental data. Note that our approach does not correct for sequencing errors in sample indexes, implying that some samples may be 'contaminated' at sequencing stage because their index is similar to that of another sample. This issue is best solved by increasing the Levenshtein distance between index sequences. It should be pointed out though that such sample index errors are not expected to affect the detection of mothers and daughter barcodes because barcode errors will occur independently of sample index errors.

Our approach is very good at distinguishing true and spurious barcodes, yet is obviously imperfect, for at least three reasons. First, a sequence that is derived by a PCR error can generate a 'granddaughter' if sequencing errors follow after the PCR mistake. These granddaughters might escape from being detected. Second, daughters of a true barcode *x* might not be detected because barcode *x* was removed due to its close resemblance to another true barcode *y*. Third, spurious barcodes which are generated by a process different from the predictable one that we describe here will escape from being detected. For example, Deakin et al. recently described high-frequent barcodes that were very different from a set of 100 known barcodes inside the sample [16]. However, given the good performance of our computational clean-up on biological samples we conclude that these mechanisms for which our strategy is not effective constitute a relatively minor source of erroneous sequences.

There are two important requirements for our computational clean-up approach to be applicable. First, it is required to sequence multiple samples (different cell types, tissues, time points etc.) within a single lane, and in which the same sequences occur at different frequencies in individual samples. We showed that 10 to 20 different samples within a lane is typically sufficient for a good performance of our approach, but this number will vary depending on the amount of overlapping barcodes between samples. Second, a sufficient sequencing depth is required to be able to observe and thus exploit the reproducibility of sequencing error rate in different samples. These requirements are typically fulfilled in cellular barcoding experiments, and rapid advancement of sequencing technology capacities will make this even more straightforward.

It will be interesting to establish whether the approach developed here can also be applied on data obtained from other sequencing platforms and for other types of sequencing data, such as TCR repertoire, cancer genome or viral variant analyses. Vice versa, methods that were specifically developed for such other sequencing data (e.g., [42, 44–48]) may potentially also be combined with our strategy for analysis of cellular barcoding data. These alternative methods are based on highly diverse approaches. For example, the algorithm ShoRAH assigns reads to clusters of haplotypes based on a Bayesian approach considering a probabilistic error rate of the sequencing process that is estimated during the analysis [42, 45, 46], PyroNoise clusters pyrosequencing data based on flowgrams rather than on the sequences [44] and EDAR and KEC exploit the frequency distributions of k-mers (substrings of reads of fixed length k) in order to find and delete or correct error regions within reads [47, 48]. To our knowledge, none of the existing error correction methods directly exploits the observation that errors across samples occur at a reproducible rate, and added value of combined approaches therefore seems realistic. A second, more general way in which our approach may contribute to the distinction of true and spurious sequences is by identifying nucleotide motifs that frequently lead to sequencing errors (as has recently been proposed to occur [49, 50]. Because our algorithm couples spurious sequences to the mother sequences from which they are derived with large fidelity, it becomes possible to analyze whether certain sequence patterns indeed lead to particular errors, including the rate at which these tend to occur.

## Conclusions

In conclusion, using cellular barcoding data with known barcodes, we demonstrated that individual sequencing error occurs at a predictable rate across samples on an Illumina HiSeq 2000 platform. Based on this finding, we devised a strategy to filter out spurious sequences, and we demonstrated that this strategy is very good at distinguishing true and spurious barcodes. Thus, our novel approach represents a useful alternative to employing an independently sequenced reference library for filtering experimental data or for cases in which sequencing such a reference library is very difficult or impossible.

### Ethics approval and consent to participate

Not applicable.

### Consent for publication

Not applicable.

### Availability of data and material

A reference implementation of the cleanup algorithm in R is included as Additional file.

## Additional files

Additional file 1: The script ‘barcodecleanup.R’ applies the clean-up procedure as described in the main text. It expects an input file consisting of a table with read counts, organized such that the columns represent the different samples and the rows represent the different barcodes. A detailed explanation of the requirements for the input file is given in the commentary of the script, and an example input file ‘testartificialdata.txt’ containing example artificial data is also provided. Barcodes containing at least one position with an ‘N’ should be removed from the input file and the barcodes should be sorted on the basis of total read count in descending order. Based on user-provided parameters (such as maximal sequence similarity between presumed correct mother-daughter pairs), the algorithm delivers two output files representing barcodes considered correct and those considered incorrect. (R 6 kb) Additional file 2: The file ‘testartificialdata.txt’ contains example artificial data to which the clean-up procedure (i.e., the script ‘barcodecleanup.R’) can be applied. (TXT 399 bytes) Additional file 3: Predictability of sequence error frequency in corresponding technical replicates. (A-D) Experiments with 19 clones of known barcodes, mixed in different frequencies and then diluted such that the expected number of cells per technical replicate is about 40 cells for all clones combined. Plots show number of reads in each of two technical replicates after normalization to 10^5^ reads in total per replicate. True barcodes are denoted by green diamonds and spurious barcodes by circles in different shades of blue and red. The exact color of the spurious barcodes represents their Levenshtein distance from one particular true barcode (highlighted in dark green-black). Note that each highly abundant true barcode generates a line of similar sequences (see different panels), strongly suggesting that these are derived by sequencing errors. (PDF 100 kb) Additional file 4: Predictability of sequence error frequency allows for detection of spurious barcodes. Log-likelihood score of presumed correct (green dots) and incorrect (red dots) mother-daughter pairs for different numbers of nucleotide differences between barcodes of a pair. Dashed line represents the threshold above which pairs are subsequently considered correct. Note that the potential to distinguish between correct and incorrect mother-daughter pairs by the log-likelihood score decreases with the number of nucleotide differences. (PDF 66 kb) Additional file 5: Impact of nucleotide difference threshold on the performance of the cleaning procedure. (A-D) The barcodes left after cleaning when using variable nucleotide difference thresholds are compared to the barcodes that are true according to the reference list of the viral barcode library. Considering the reference list as a gold standard, the sensitivity, specificity and precision are shown for each of the four individual lanes. Note that sensitivity declines with increasing threshold, whereas specificity and precision increase. (PDF 29 kb) Additional file 6: Impact of the offset of the log-likelihood threshold on the performance of the cleaning procedure. (A-D) The barcodes left after cleaning for variable log-likelihood offset thresholds are compared to the barcodes that are true according to the reference list of the viral barcode library. Considering the reference list as a gold standard, sensitivity, specificity and precision are shown for each of the four individual lanes. Dashed vertical lines denote the default value for the offset. Note that sensitivity declines with increasing offset, whereas specificity and precision increase, and the strongest effect is for large values of the offset. (PDF 32 kb) Additional file 7: Impact of the number of samples in a sequencing lane on the performance of the cleaning procedure. (A-D) The barcodes left after cleaning when using variable sample numbers are compared to the barcodes that are true according to the reference list of the viral barcode library. Considering the reference list as a gold standard, sensitivity, specificity and precision are shown for each of the four individual lanes. Note that especially precision benefits from large sample numbers. (PDF 34 kb)

## Abbreviations

LMPP: lymphoid-primed multipotent progenitors
NGS: next generation sequencing
TCR: T cell receptor
