# Supplementary figures and images for: Reproducibility of Illumina platform deep sequencing errors allows accurate determination of DNA barcodes in cells
Source: BMC Bioinformatics. 2016 Apr 2;17:151. doi: 10.1186/s12859-016-0999-4 (PMC4818877; doi:10.1186/s12859-016-0999-4)

# Additional File 1

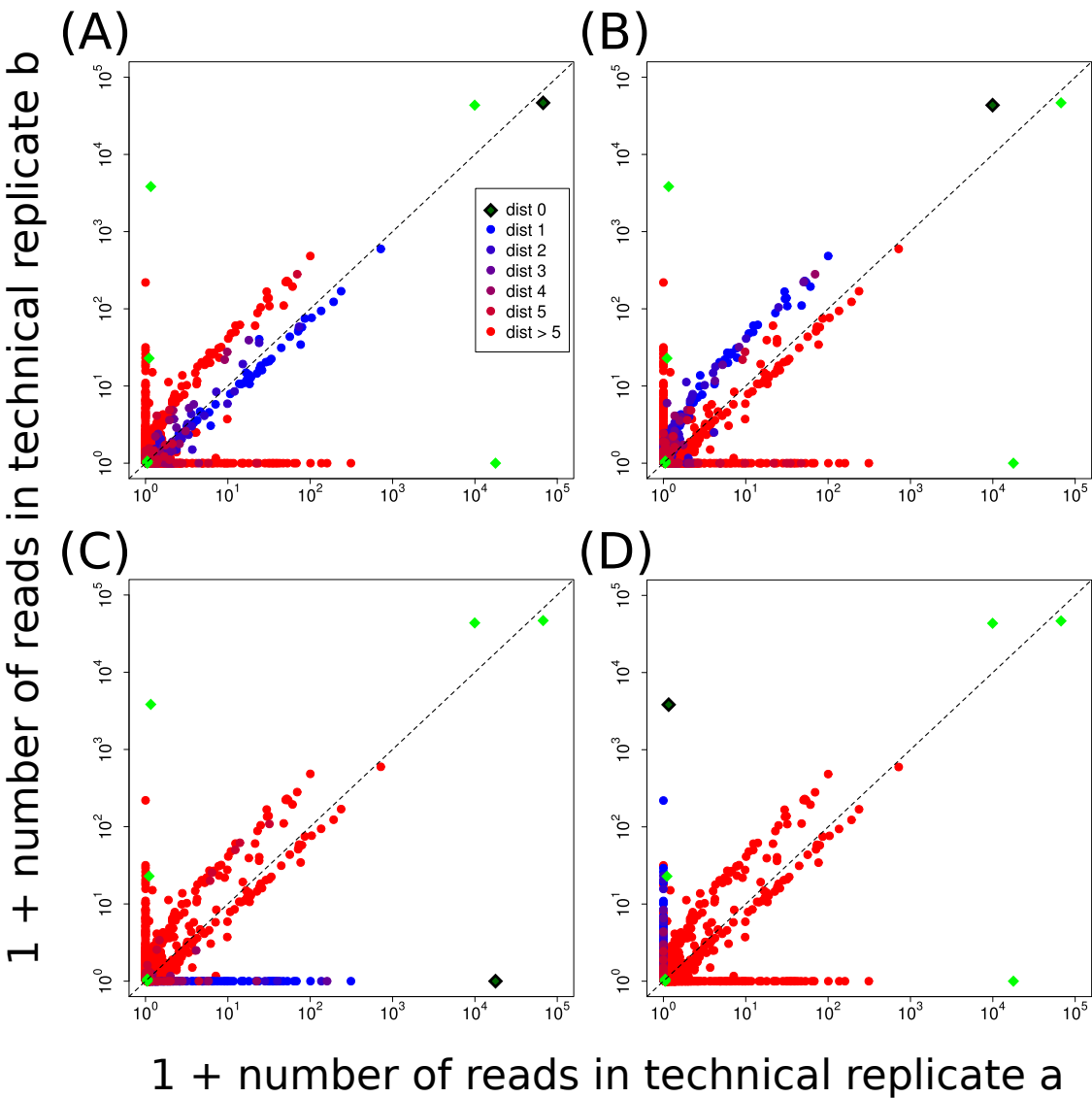

Supplement: Additional file 3: — Predictability of sequence error frequency in corresponding technical replicates. (A-D) Experiments with 19 clones of known barcodes, mixed in different frequencies and then diluted such that the expected number of cells per technical replicate is about 40 cells for all clones combined. Plots show number of reads in each of two technical replicates after normalization to 105 reads in total per replicate. True barcodes are denoted by green diamonds and spurious barcodes by circles in different shades of blue and red. The exact color of the spurious barcodes represents their Levenshtein distance from one particular true barcode (highlighted in dark green-black). Note that each highly abundant true barcode generates a line of similar sequences (see different panels), strongly suggesting that these are derived by sequencing errors. (PDF 100 kb) [file 12859_2016_999_MOESM3_ESM.pdf]

# Additional File 2

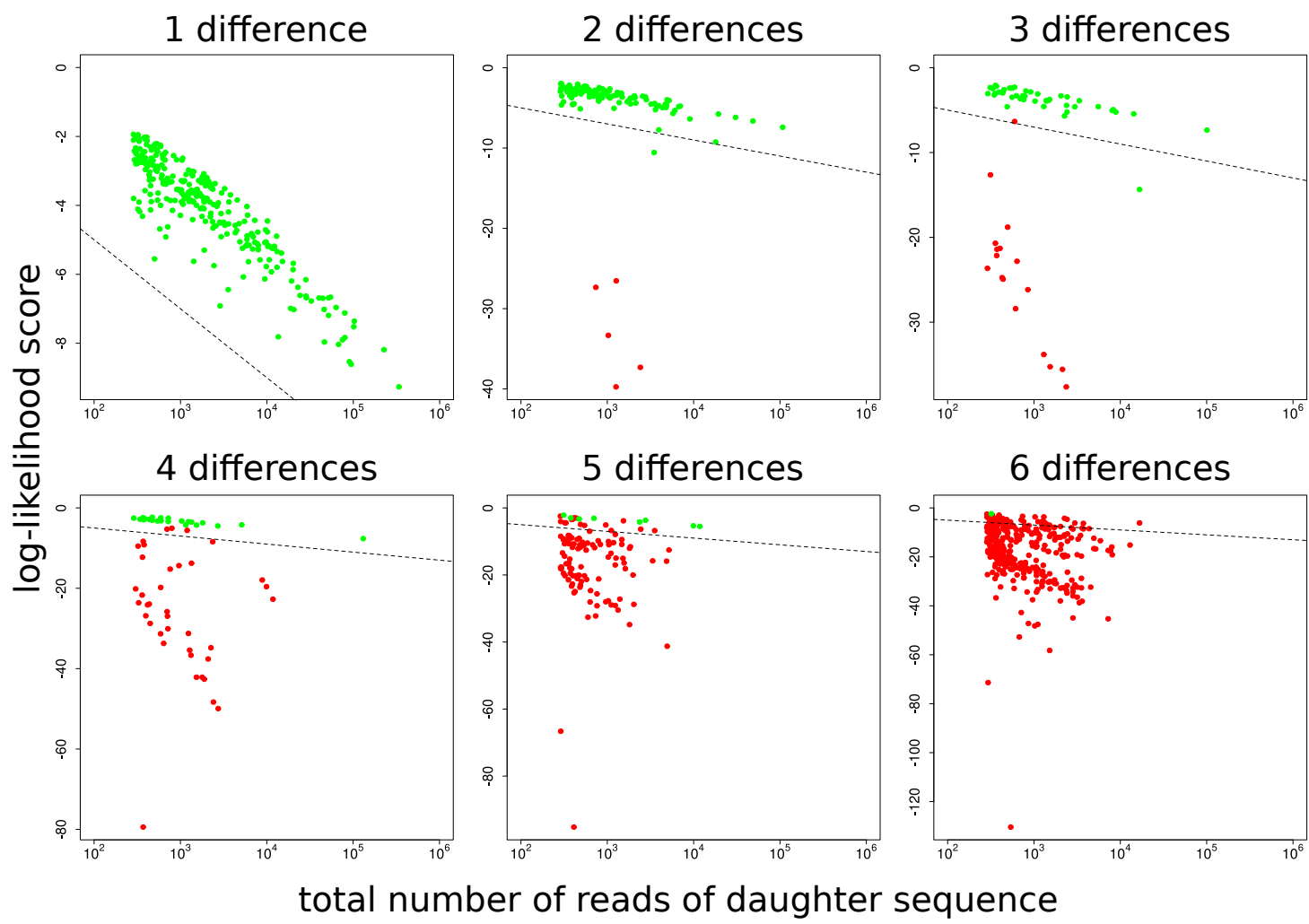

Supplement: Additional file 4: — Predictability of sequence error frequency allows for detection of spurious barcodes. Log-likelihood score of presumed correct (green dots) and incorrect (red dots) mother-daughter pairs for different numbers of nucleotide differences between barcodes of a pair. Dashed line represents the threshold above which pairs are subsequently considered correct. Note that the potential to distinguish between correct and incorrect mother-daughter pairs by the log-likelihood score decreases with the number of nucleotide differences. (PDF 66 kb) [file 12859_2016_999_MOESM4_ESM.pdf]

# Additional File 3

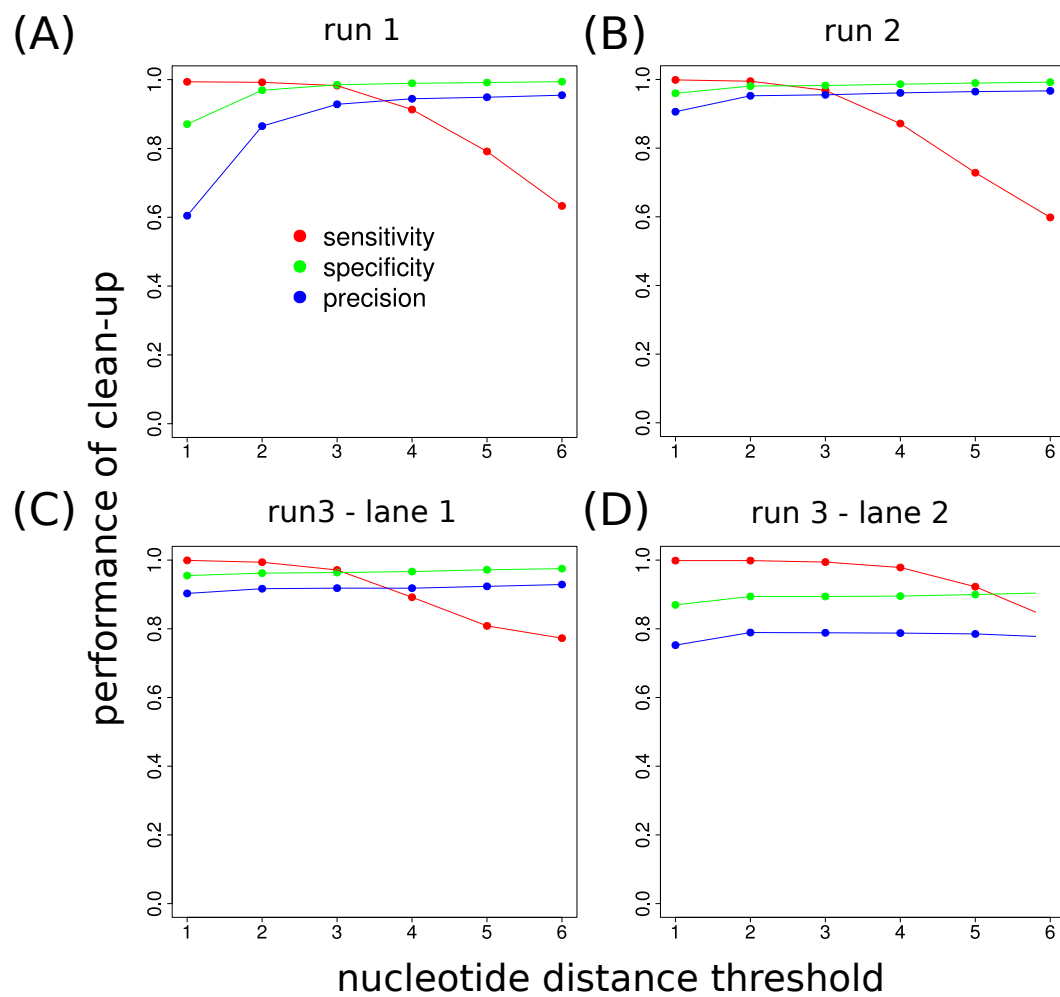

Supplement: Additional file 5: — Impact of nucleotide difference threshold on the performance of the cleaning procedure. (A-D) The barcodes left after cleaning when using variable nucleotide difference thresholds are compared to the barcodes that are true according to the reference list of the viral barcode library. Considering the reference list as a gold standard, the sensitivity, specificity and precision are shown for each of the four individual lanes. Note that sensitivity declines with increasing threshold, whereas specificity and precision increase. (PDF 29 kb) [file 12859_2016_999_MOESM5_ESM.pdf]

# Additional File 4

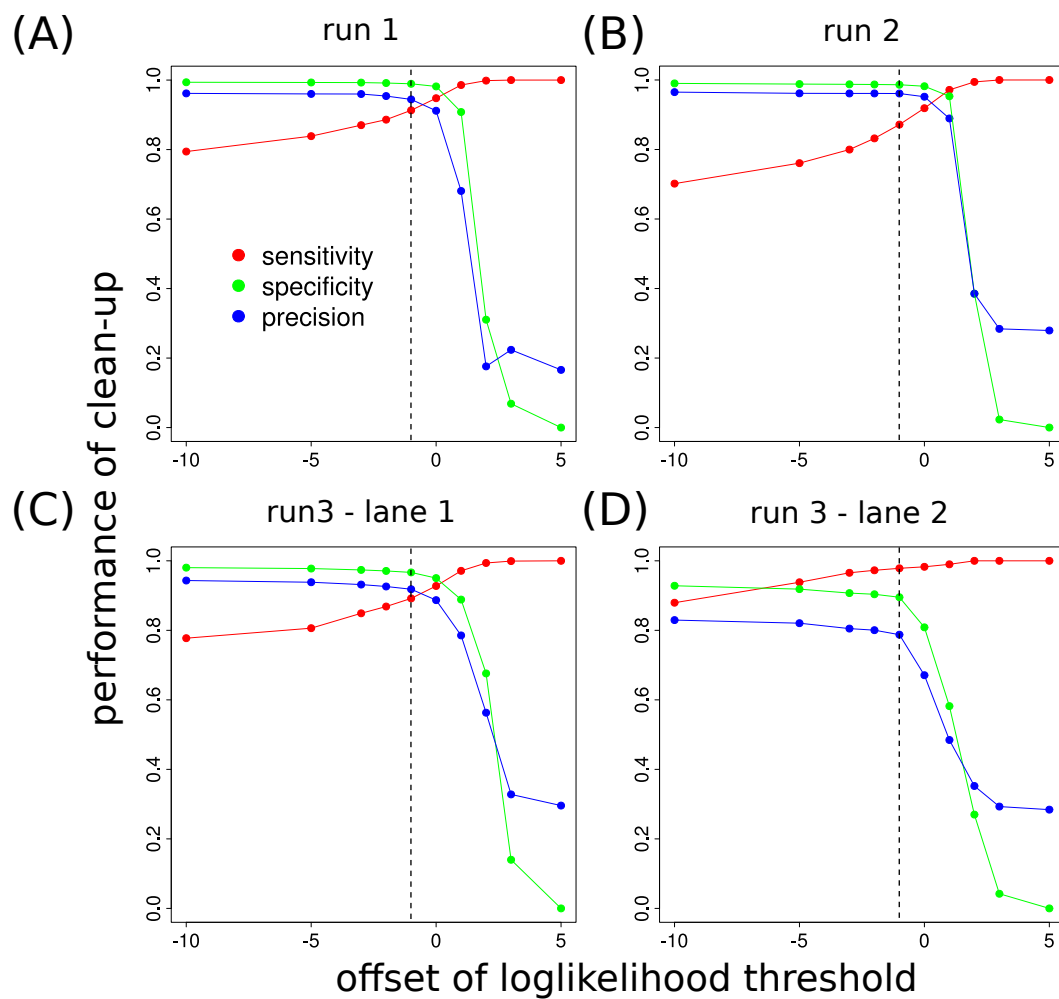

Supplement: Additional file 6: — Impact of the offset of the log-likelihood threshold on the performance of the cleaning procedure. (A-D) The barcodes left after cleaning for variable log-likelihood offset thresholds are compared to the barcodes that are true according to the reference list of the viral barcode library. Considering the reference list as a gold standard, sensitivity, specificity and precision are shown for each of the four individual lanes. Dashed vertical lines denote the default value for the offset. Note that sensitivity declines with increasing offset, whereas specificity and precision increase, and the strongest effect is for large values of the offset. (PDF 32 kb) [file 12859_2016_999_MOESM6_ESM.pdf]

# Additional File 5

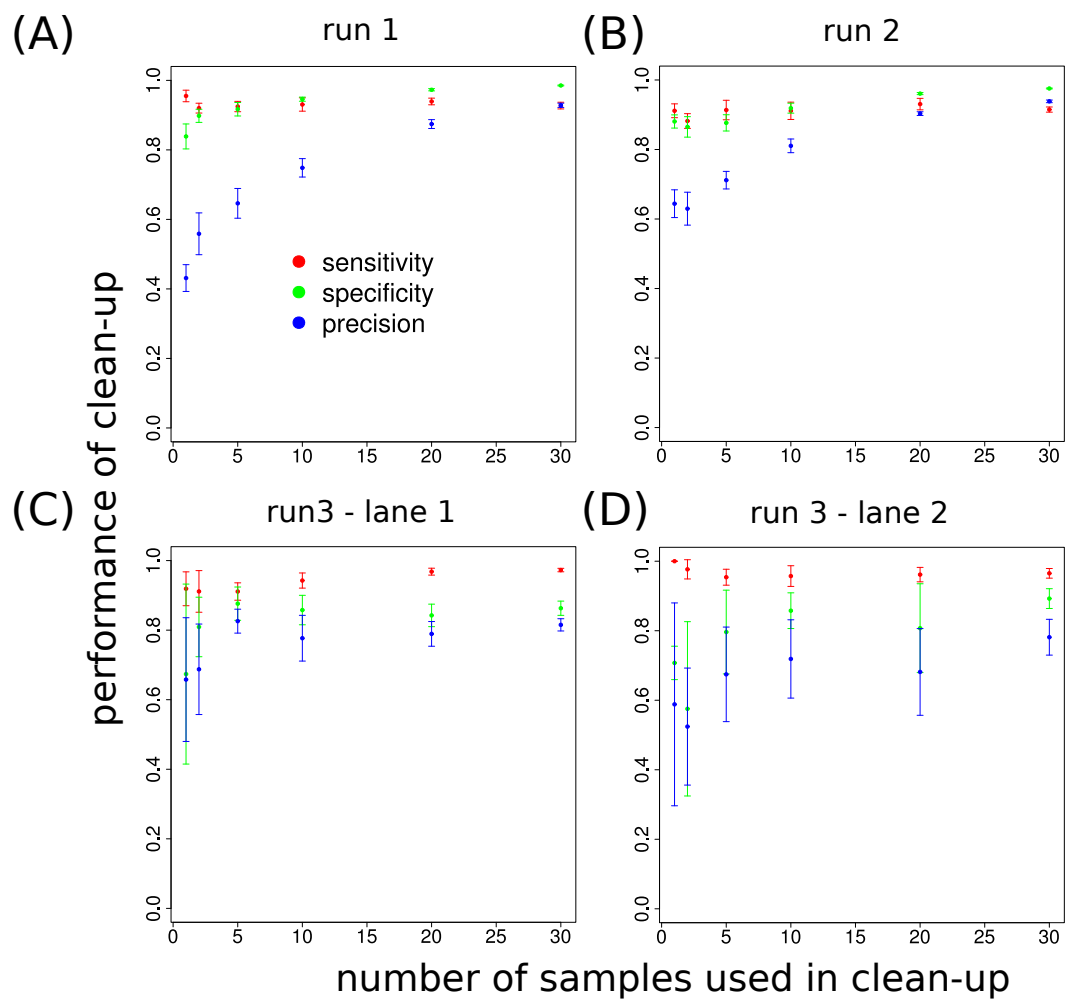

Supplement: Additional file 7: — Impact of the number of samples in a sequencing lane on the performance of the cleaning procedure. (A-D) The barcodes left after cleaning when using variable sample numbers are compared to the barcodes that are true according to the reference list of the viral barcode library. Considering the reference list as a gold standard, sensitivity, specificity and precision are shown for each of the four individual lanes. Note that especially precision benefits from large sample numbers. (PDF 34 kb) [file 12859_2016_999_MOESM7_ESM.pdf]
